# Community‐dwelling older adults' acceptance of smartwatches for health and location tracking

**DOI:** 10.1111/opn.12490

**Published:** 2022-07-12

**Authors:** Jane Chung, Heidi Rishel Brakey, Blaine Reeder, Orrin Myers, George Demiris

**Affiliations:** ^1^ Virginia Commonwealth University School of Nursing Richmond Virginia USA; ^2^ Clinical and Translational Science Center University of New Mexico Albuquerque New Mexico USA; ^3^ University of Missouri School of Nursing Columbia Missouri USA; ^4^ University of Missouri Institute for Data Science & Informatics Columbia Missouri USA; ^5^ Department of Family and Community Medicine, School of Medicine University of New Mexico Albuquerque New Mexico USA; ^6^ University of Pennsylvania School of Nursing Philadelphia Pennsylvania USA

**Keywords:** activity trackers, GPS tracking, location tracking, older adults, smartwatches, technology acceptance

## Abstract

**Background:**

Despite rapid growth in the popularity of smartwatches, evidence lacks regarding older adults’ acceptance of smartwatches. Since most wearable sensors are not designed specifically for older adults, there is a need to examine wearability and usability challenges of wearable sensing devices faced by older adults to facilitate the use of objective measurements of health and mobility.

**Objectives:**

We aimed to examine older adults' perceptions of GPS‐enabled smartwatches and to identify potential barriers and facilitators of smartwatch and sensor data use.

**Methods:**

As part of a larger feasibility study, we conducted a mixed‐methods study that included a descriptive content analysis of interviews and a brief usability survey with 30 participants aged 60 years and older after they had used a smartwatch for 3 days.

**Results:**

Most participants perceived wearable activity trackers including smartwatches and sensor‐based data as useful for tracking health, finding activity patterns and promoting healthy behaviours. Privacy was of little concern, leading to willingness to share activity and location data with others. Participants identified barriers to usability as clumsy design, lack of aesthetic appeal, and difficulty reading the display and using the GPS tracking function. In contrast, identified facilitators of adoption included a big display, high‐tech look, self‐awareness and possible behaviour change.

**Conclusions:**

Smartwatches have the potential of personalised detection of health deterioration and disability prevention, based on analysis of older adults' activities in free‐living environments. The usefulness of this technology for older adults can be significantly increased by addressing usability issues and providing instructions on challenging features.

**Implications for Practice:**

To support sustained self‐monitoring behaviours through wearable sensor devices in older adults, it is critical to examine how they perceive those devices and identify factors affecting technology acceptance that can maximise adoption.

**What does this research add to existing knowledge in gerontology?**

- This study suggests that wearable health monitoring devices need to be designed to meet the needs, preferences, and use context of older adults.
- Several physical characteristics of wearable sensing devices are identified as barriers to device adoption and continued use, such as design, size, complexity, battery life and easiness of display reading.

**What are the implications of this new knowledge for nursing care with older people?**

- Technology‐based nursing interventions aimed to accurately assess health status and deliver preventive care to older adults need to focus on understanding barriers to technology use in the everyday living context.
- Nurses should consider the older adult user's prior technology use, health literacy, self‐efficacy and availability of technology support when designing technology‐enabled health care.

**How could the findings be used to influence policy or practice or research or education?**

- There should be an effort to identify facilitators of and barriers to sustained self‐monitoring behaviours among older adults that are unique to the sociocultural context of use.
- Our research warrants continued privacy research as it relates to health and location tracking to ensure optimal remote monitoring consistent with user values of privacy, independence and autonomy.

## 1 INTRODUCTION

The increasing number of older people globally highlights the need for interventions and tools to help this group become engaged in health promotion, managing symptoms and identifying early signs of illness. Additionally, health care is moving beyond clinical settings into homes where older individuals must take an active role in their health care (Adler‐Milstein et al., 2017). Mobility is important to healthy ageing, with implications for independence and quality of life (Webber et al., 2010). One way to understand mobility is to frame it within the concept of ‘life space’, defined as the size and pattern of the area in which individuals move within their environments (Baker et al., 2003; Peel et al., 2005). Accurate and frequent measures to detect early mobility changes in older adults are indispensable in the identification of functional deterioration that may result in safety risks. Traditionally, mobility assessments occur during sporadic, brief clinic visits, and depend on self‐reported events (Lyons et al., 2015). These methods may be biased or unreliable and do not capture within‐day or day‐to‐day variations. Given significant gaps in the assessment and measurement of mobility among community‐dwelling older adults (Petersen et al., 2015; Prohaska et al., 2011), there is a need for reliable and valid measures to identify those at risk for safety and adverse health outcomes associated with changing mobility status.

The use of miniaturised body‐worn sensing devices incorporating motion and physiological sensors has gained attention by researchers and clinicians because they may benefit users by tracking various health indicators (Kikhia et al., 2016; Quiroz et al., 2018; Straiton et al., 2018). This technology provides an opportunity to collect and reflect on one's activity and health status and thus motivate oneself to engage in healthier behaviours (Seifert et al., 2017). Further, these devices have the potential to enable more effective communication among patients, caregivers and healthcare providers by transmitting basic vital information or providing features that lead to better coordination of care and possibly improved health outcomes (Demiris et al., 2013). Sensor‐based activity and health tracking, combined with advances in big data analysis, also have the potential to extend the scope of precision medicine by generating personalised information based on physical activity, physiologic, environmental and lifestyle data, comparing it with genetic and biologic data (Brennan & Bakken, 2015; Collins & Riley, 2016).

Of particular interest is the fusion of motion sensing (e.g. accelerometers, gyroscopes) and location tracking in wrist‐worn activity trackers. Location tracking allows continuous recording of location coordinates and time and can be used to quantify mobility‐related measures, such as gait speed, distance moved, and movement path and size (Bayat et al., 2021; Mahendran et al., 2016). Thus, commercially available wearable global positioning system (GPS) devices could be used as an alternative for more accurate and frequent data to detect abnormal patterns or changes in physical activity and mobility that may indicate underlying diseases or functional deterioration. With the introduction and use of GPS‐enabled activity trackers, it is now possible to collect objective data to characterise and measure mobility and travel behaviours in free‐living environments (Bayat et al., 2021; Boissy et al., 2018).

Despite these potential benefits, the older adult population lags behind in wearable activity tracker adoption and use. One study identified only 7% of those 65 years or older owned an activity tracker in 2013 (Ledger & McCaffrey, 2014). Another found only 8.5% of individuals in Switzerland aged 65–79 years and 6.2% of those 80 years or older used an activity tracker; 6.8% and 2.3% in these respective age groups used a smartwatch for physical activity tracking (Seifert et al., 2017). This population may lack experience using mobile devices or wearable sensors (Pew Research Center, 2017), which may prevent them from fully understanding the technical information they receive. Further, most wearable devices are not specifically designed for older adults. Ageing‐related changes in perceptual, cognitive and motor skills affect older adults' use of small devices (Charness & Boot, 2009). Potential harm and abuse from privacy invasion and breach of sensitive information are also possible (e.g. identity theft or financial crimes) (Chung et al., 2017).

According to the Technology Acceptance Model (TAM), understanding factors related to user satisfaction and acceptance is critical to prediction and explanation of one's willingness to purchase or adopt technology‐supported solutions (Davis, 1989). While research on adoption and user challenges are in abundance, few have examined older adults' acceptance of GPS‐enabled activity trackers and factors contributing to their decision to use those trackers in daily life. Preusse et al. (2017) identified several factors related to perceived usefulness and perceived ease of use of two activity trackers among older adults, including visibility of system status (e.g. battery levels or device sensitivity setting), simplicity of tracker presentations, goal‐setting functions, perceived inaccuracy, necessary time and effort, and easy‐to‐use formats. Puri et al. (2017) found older adults were concerned about being forgetful to use, losing and inconvenience of carrying the device, while they appreciated goal setting and motivational features. Several physical characteristics of activity trackers were identified as influencing device acceptance, such as display, battery life, aesthetics and comfort. Another study examined the usability of four commercially available activity trackers and one pedometer with adults aged over 50 (Mercer et al., 2016). Participants perceived the devices were useful especially for behaviour change, while they desired to use a tracker with a clear display developed specifically with and for older adults, with instructions provided. Similarly, Kononova et al. (2019) reported that older adults preferred activity trackers that are aesthetically appealing, comfortable, waterproof and easy to use.

The literature provides insight into the significance of wearability and usability in older adults' acceptance and continued use of activity trackers for health monitoring. However, previous studies are limited to simple fitness bands, mostly accelerometer‐based technology. A growing number of people looking to track fitness and health are choosing wearable devices with built‐in *GPS and complex tracking functionality, such as smartwatches*. These are usually bigger in display and band size compared to simple fitness trackers due to the GPS receiver requiring a larger battery size, which might lead to unique wearability and usability challenges by older adults (Boissy et al., 2018). This could affect user compliance and data accuracy and reliability.

To date, few studies have explored older adults' perceptions of smartwatches with a GPS function (Hardy et al., 2018). There is great potential in building an understanding of this type of technology that can facilitate objective measurements of mobility and physical function among older adults. The purpose of this study was to explore attitudes and perceptions of community‐dwelling older adults towards smartwatches with location tracking and the use of sensor data using a mixed‐methods approach.

## 2 MATERIALS AND METHODS

### 2.1 Design

This was part of a larger feasibility study to understand functionalities of smartwatches, and location data from these devices, for measuring life‐space mobility among older adults. Participants wore the device for 3 days, after which we conducted a mixed‐method analysis of interviews to examine their overall evaluation. Mobility assessment outcomes have been published elsewhere (Chung et al., 2022). The study protocol was approved by the Institutional Review Board at the University of New Mexico (UNM). We evaluated the quality of the study using the Consolidated Criteria for Reporting Qualitative Research checklist (Appendix S1; Tong & Sainsbury, 2007).

### 2.2 Device

We used the Fitbit Surge, a smartwatch with location tracking and other activity‐tracking functions (e.g. steps, floors climbed, distance moved, heart rates) to collect life‐space mobility data. This device is rechargeable and can be worn like a watch. Motion sensors are embedded in the device, including a 3‐axis accelerometer, gyroscope, altimeter and GPS. It has a battery life of approximately 10 h with GPS on and weighs 32 g.

### 2.3 Participants

We used convenience and snowball sampling for recruitment. We presented the study to internal medicine clinicians at UNM and posted and emailed flyers to healthcare clinics, community centres, senior living communities, and personal and professional networks. Interested parties contacted the study team to determine eligibility. Participants were required to be 60 years or older, speak and read English, not mostly bed‐ or wheelchair‐bound, be willing to use a Fitbit Surge for 3 days with GPS tracking, and be willing for data to be uploaded to the Fitbit website and shared with the research team. One of the reasons to choose the sample with an age criterion over 60 years was to have a broad enough pool of participants to find the relationship between life‐space mobility and cognitive function in the parent study, given that age‐related cognitive decline begins before age 60 (Salthouse, 2009). No relationship was established prior to study commencement, except four participants were acquaintances of the interviewer. The team reviewed and approved of this relationship as one that would not introduce bias, prior to enrolling participants, since this was a pilot study with strict time constraints with the purpose of informing a larger clinical trial. Additionally, the PI who did not know these participants reviewed the transcripts of the interviews to ensure impartial questioning and adherence to the interview protocol prior to inclusion for analysis.

### 2.4 Data collection

One visit at baseline and one at exit occurred at a chosen location by each participant (e.g. their home, community centre; May through July 2016), and no one else was present besides the participant and interviewer. Visit one was 30–60 min and consisted of informed consent and questionnaire administration. Written informed consent was obtained from the participant before the baseline examination. Participants received written instructions with pictures, and we ensured they knew how to use the device before leaving. They were instructed to wear the smartwatch with GPS on, from approximately 8 am to 6 pm for 3 days. The exit visit generally occurred one week after baseline and lasted approximately 30 min. A study coordinator (HRB) conducted a survey and interview to assess (1) general attitudes towards sensor‐based activity monitoring; (2) perceived usefulness and ease of use; (3) access and perceived usefulness of sensor data; and (4) concerns related to smartwatches with location tracking. Interview questions were informed by TAM (Davis, 1989) and previous studies (Chung et al., 2017; Reeder et al., 2013) and included four usability questions on a 7‐point Likert scale, regarding device display, functions, attractiveness and ease of use (see Table S1 for interview questions). Participants returned the device and received $100. Upon completion of activity monitoring, we downloaded accumulated GPS records from the Fitbit website.

### 2.5 Data analysis

This manuscript examines demographic, usability survey and in‐depth interview data from the exit visit. We used descriptive statistics to analyse the demographic and usability data (mean ± standard deviations, *n*, and %). Audio recordings of interviews were professionally transcribed and imported into NVivo 10 qualitative analysis software (QSR International) for coding. We conducted a content analysis using a systematic iterative process (Graneheim & Lundman, 2004; Polit & Beck, 2008). The coding team consisted of the principal investigator (PI; JC) and qualitative analyst (HRB). The PI is a PhD‐trained nurse researcher and has over a decade of mixed‐methods research experience in the areas of ageing, mobility, and technology design and evaluation. Another member is a master's‐level research specialist with 9 years' experience in various health science mixed‐methods research. They jointly deductively generated codes based on interview questions and TAM, to which they independently coded five transcripts. As categories arose, they inductively created new codes and grouped similar codes under categories. They met to discuss coding and reconcile disagreements until reaching consensus. Upon agreement, one analyst (HRB) coded the remaining transcripts. They continued to meet regularly to discuss new categories, revise the coding structure as needed (see Appendix S2 for final codebook), and determined they reached data saturation. Finally, they jointly reviewed quotes within each category and further coded to refine and summarise findings.

Ensuring the rigour of the qualitative analysis process was based on the use of two reviewers and the consensus achieved in team meetings. The consensus was reached with the addition of three new codes to the original codebook, while the categorizations remained intact. An audio trail to capture thought processes and decisions for coding was kept.

## 3 RESULTS

Here, we report descriptive statistics obtained through the demographic questionnaire and quantitative questions first, and then report qualitative findings.

### 3.1 Quantitative results

#### 3.1.1 Demographic characteristics

Thirty older adults participated in the study (mean age 67.4, 63% female). Twenty‐one (70%) had more than a bachelor's degree. Fifty‐three per cent were married or partnered. Two‐thirds of participants had one or more chronic conditions, such as arthritis, hypertension or diabetes mellitus (Table 1).

**TABLE 1:** Participant demographics and clinical variables (*N* = 30)

Variable	M (SD), range	*n* (%)
Age	67.4 (5.3), 60–79	
Female		19 (63)
White		23 (77)
Hispanic or Latino, n (%)		6 (20)
Education		
High school diploma/GED		2 (7)
Some college		7 (23)
Bachelor's degree		6 (20)
Graduate or Professional degree		15 (50)
Married/partnered		16 (53)
Health condition		
Arthritis		14 (47)
Hypertension		9 (30)
Diabetes mellitus		7 (23)
Cancer history		7 (23)
Assistive device use		4 (13)

#### 3.1.2 Usability questionnaire data

When asked how appealing the device was overall, on a scale of one (not appealing) to seven (very appealing), the mean answer was 5.05 (SD = 1.85). Most were satisfied with the display (M = 5.59, SD = 1.68) and its functions (M = 5.64, SD = 1.76). Overall, participants thought the device was easy to use (M = 5.5, SD = 1.58, Table 2).

**TABLE 2:** Participant perceptions of the device and data sharing

Question item	M (SD)	*n* (%)
Usability questions		
How satisfied with the display of the device?^a^	5.6 (1.7)	
How satisfied with the function?^a^	5.6 (1.8)	
How appealing was the device overall?^a^	5.1 (1.8)	
How easy was it to use?^a^	5.6 (1.6)	
Quantification of responses to Yes/No questions		
Sharing data with healthcare providers, family or friends (yes)		24 (80.0)
Has having this device changed the way you carry out your daily activities? (No)		23 (76.7)
Would you like to be able to turn on or off the device depending on your preference? (yes)		15 (50.0)

*Note:* Higher scores indicate higher levels of satisfaction, attractiveness, and perceived ease of use.

^a^ Score range: 1–7.

### 3.2 Qualitative results

#### 3.2.1 Older adults' acceptance of wearable activity trackers

Participants discussed usefulness of wearable activity trackers and were generally interested in them for various reasons. The potential value for measuring indicators of health (e.g. heart rate, blood pressure and diabetes) or physical activity levels was discussed. Perception of health seemed to influence potential usefulness of the technology. For example, one person with atrial fibrillation appreciated its heart rate monitoring function. However, participants who perceived themselves as healthy or physically active did not feel the need to use activity sensors. Nevertheless, its potential to motivate people to move more was mentioned. One person discussed its novelty for his age, ‘*I think it's helpful for us older people because we've never been able to have something like this to help get us motivated and move* (P26)’. Another commented that if he could monitor his own health indicators, the device could potentially replace or prevent a medical or emergency department visit.

Participants did not seem to be fully aware of the watch's capability, thereby leading to decreased interest in smartwatch adoption. Older adults stated activity trackers would not be useful nor encourage healthier habits because functions seemed limited to tracking steps and heart rates without more in‐depth health information. One stated, ‘*I just think that's dumb. If you have to rely on [it] to tell you how many calories you burned…and how many steps you've taken, you're really lacking something in your life* (P4)’. Mixed responses about whether they liked its functions were also observed.

The interview data reveal that usefulness is dependent on lifestyle. For example, if the technology did not support activities they usually engage in (e.g. swimming, biking, and dancing), they were unlikely to adopt it. Participants mentioned additional aspects that could influence their decision to use any wearable activity tracker, including peer or healthcare provider endorsement, previous technology experience and exposure, and general interest in technology.

#### 3.2.2 Physical characteristics of the device matter

Participants reported several physical characteristics of the device that made them feel uncomfortable or were perceived as less appealing. The uncomfortable features include a bulky design, device size and material. Participants expressed that the watch strap bands negatively impacted their use, ‘*I had a little trouble with it. When I was riding the bike, it was sticky. It kind of stuck to my skin* (P7)’. Women especially complained about its size and aesthetics, ‘*It's not the most attractive thing if you dress up* (P23)’. On the contrary, people expressed their satisfaction with its tech‐savvy look, ‘*I did enjoy wearing it because it's something you can flaunt* (P22)’.

Participants confirmed the design of display and visibility had a significant influence on acceptance. For example, older adults had a hard time seeing it in lower light, the backlight was difficult to turn on, and, ‘*it glares too much in the daylight* (P4)’. One common complaint was about the amount of space the stopwatch took on the screen, which automatically turned on with GPS tracking. Even though participants did not like its overall size, they seemed to like the big display: ‘*I can see it without my glasses, so that was great* (P22)’. The touch screen function was mentioned as a feature that made the device use easier.

Additionally, some concerning aspects of the device were found, including the optical heart rate sensor. ‘*It was kind of creepy putting those blinking lights on my wrist*… *What is it doing to me? How deep is it going? Is radiation getting in there*? (P2)’ Similarly, one person commented that the electrical field would impact her body, causing a harmful effect on health. Additionally, people would have liked it to be waterproof with longer battery life and technical reliability of device operations.

#### 3.2.3 Desire for easy‐to‐use devices and need for instructions

Participants provided specific examples of when they felt difficult to use the device. For example, there was a comment that turning on the GPS function was somewhat difficult to execute initially but became easier over time. Also, participants said they still needed to reference instructions for help: ‘*I was surprised when you gave me the instructions. I read through them a couple times*…(P29)’. Sometimes it was not easy to check a certain type of data on the display: ‘*…by the time you finally get to it and scoot through all the little windows, your heart rate has now changed, so what good is that?* (P4)’ One person noted, ‘*If I [knew] which display would cover what functions, then I would have appreciated it a little bit more* (P9)’. Also, one's confidence of previous technology experience seemed to affect perceived ease of use.

#### 3.2.4 Perceptions of sensor‐based activity and health data

Participants generally held positive views of personal activity and health data collected by the device. They were satisfied with the display showing calories, steps and heart rates. However, a desire to learn about the meaning of those values in relation to their health was expressed. One participant stated, ‘*It says I went up 29 floors. What does that mean in English? […] Yesterday, I burned almost 3,000 calories by 5:00. What does that translate into?* (P4)’ Participants wanted to see data from their activity monitoring. Desired frequency of data access varied greatly (Table 3). However, we observed hesitancy to check the data because ‘*It would be disappointing* (P16)’.

**TABLE 3:** Desired frequency of data access and types of data participants wished to see

Item	Number of participants selecting
Desired frequency of data access	
Never	2
Daily	17
Weekly	6
Monthly	1
Seasonal/Quarterly	1
Depends	1
Once	2
Data types interested^a^	
Heart rate	18
Steps and distance moved	17
Calories	11
Blood pressure	5
Summary of overall activities	4
Sleep	2
Oxygen level	1
Stairs climbed	1

^a^ Multiple selections were possible.

Participants identified potential uses for these data, such as monitoring level of physical activity, finding certain periods of the day for scheduling exercise, generating an alarm about a subtle change in sleep or activity patterns, tracking diet and monitoring health‐related indicators. One participant said the heart rate monitoring function was especially useful because he had had open‐heart surgery. Additionally, participants commented it would help to compare their activity level with the level of others, ‘*To see where I'm at with people of my own age* (P4)’, or for motivating and supporting each other.

Participants shared what types of data they were most interested in seeing from activity sensing devices. They were most interested in seeing heart rate and steps/distance data (Table 3). People wished the device was capable of monitoring blood pressure or oxygen saturation levels as an indicator of their cardiac functions.

#### 3.2.5 Willingness to share sensor‐based data with others

Participants were willing to share personal location and activity data with healthcare providers for monitoring health and activity status. One person commented, ‘*It would contribute to their understanding of my state of health… If they had to monitor some illness or disease, I'd imagine it'd be useful information* (P23)’. Another said data sharing could be used as a way to show, ‘*I am trying to follow [my provider's orders]* (P24)’ or help providers ‘*to motivate other patients* (P29)’. The interviews reveal several factors that might prevent them from sharing data, such as distrust in healthcare providers, provider lack of interest, concerns about inappropriate data use or sharing, or potential embarrassment due to low activity levels.

Participants expressed willingness to share activity data with family members because they wanted them to know ‘*I'm healthy or something* (P6)’, or motivate others to change health behaviours. One woman wanted to compare her activity with her husband's or sister's. Another cited concern about deteriorating health as she aged as a reason for sharing. Reasons for not wanting to share data with family were provided: ‘*I wouldn't share it with my family… Because mostly, I don't think everybody needs to know how active I am* (P30)’. Another said she would not share with family because they live far away and would prevent them from being actively engaged in a caregiving role if they could know her activity level.

#### 3.2.6 Using activity trackers as a tool for self‐awareness and behaviour change

Participants were generally interested in using activity trackers to assess their own activity levels. The device made them more aware of their activity level and realised they need to move more, ‘…*Because you can't exaggerate on how far your run was. I always thought it was six miles, but it's like four and a third or something* (P3)’. Participants reported using the device influenced them to increase physical activity. Stars or badges displayed for certain goals valuable to maintain healthy behaviours: ‘*Oh, a star. I need to get that star today* (P18)’.

There was a comment that simply using the device would not make any change in one's activity levels. Reasons for no resulting behavioural change were provided, such as a short period of using the device, a desire to keep activities exactly the same and a temporary medical limitation. Interestingly, one person was motivated to do less exercise after seeing her data, ‘… *I'm pretty active as it is, but maybe it made me more thoughtful to maybe try to rest once in a while and slow down* (P25)’.

#### 3.2.7 Privacy concerns related to activity and location tracking

When asked about privacy, people had mixed opinions. Overall, participants reported no major privacy issues because they thought activity trackers collect simple health data without much meaning, ‘*If somebody wants to invade that and see how many beats I have per minute, that's fine. It's not that personal* (P9)’. Nevertheless, participants noted that the device created a feeling of being watched ‘*If I [had one], I would feel more like somebody is watching me. I don't need anybody to watch me to see whether I'm going to exercise* (P7)’. There were concerns about ‘*Big Brother* (P28)’ and unauthorised information access.

Regarding location tracking, there were mixed responses. One person commented, ‘*As long as it wasn't recording my conversations, I was fine with that* (P11)’. Another stated he might not be worried, ‘*because even if somebody got it, they wouldn't know who* (P9)’. However, participants worried about unexpected consequences resulting from the location being exposed to an unauthorised person: ‘*They know you're not home, so they can rob your house, or steal your car* (P5)’.

## 4 DISCUSSION

We found most participants were generally positive towards the idea of wearable activity trackers, including smartwatches with location‐tracking features, to measure health indicators and for motivation to increase physical activity. Many thought this technology could help them be healthier and possibly reduce medical visits. Especially older adults with a chronic illness or facing health declines expressed their desire to adopt any type of activity tracker for health monitoring. We identified a need to design wearable devices with the ability to track a wider range of activity (e.g. swimming and dancing) and health indicators (e.g. blood pressure and oxygen saturation). This is consistent with literature showing older adults expected a device with a wider variety of functions (Kononova et al., 2019) and that end‐user needs and perceived health status are key factors determining wearable sensor technology adoption and use (Ehn et al., 2018; Nguyen et al., 2017; Puri et al., 2017).

This study revealed several physical characteristics of the device as barriers to adoption, such as design and size, complexity, short battery life, lack of waterproof design or difficulty reading the display with reflections or in low light. Although advanced wearable sensing devices (smartwatches) provide a novel opportunity to improve health and wellness, older adults may not use them because of usability and wearability issues. Previous studies have evaluated the use of smartwatch location tracking in supporting physical activity for this population, particularly in the collection of data for mobility research (Mahendran et al., 2016; Storey et al., 2013; Wieters et al., 2012). Increasing attention has been given to the issue of older adults' acceptance and continued use of health and wellness management technologies. The users' preferences and needs must be explored, for example, factors affecting usability and context of use (Peek et al., 2016). Similar to previous studies (Ehn et al., 2018; Hardy et al., 2018; Puri et al., 2017), we found size and aesthetics are an important consideration for smartwatches. For example, most participants (especially women) thought the device was ‘too bulky’. Fitness watches are usually bigger than simple fitness trackers due to the need for longer battery life and advanced behavioural sensing capabilities. This suggests a need for co‐designing new solutions with older adults to elicit ideas about what features are preferable and how the design can support their need for behaviour change, including self‐monitoring of target health indicators, goal setting, behaviour feedback, and motivating oneself and others (Lyons et al., 2014). Additionally, there should be considerations of individual characteristics and social factors (e.g. access, prior technology experience, technical support, social influence) that could affect older adults' attitudes towards, acceptance of, and continued engagement with health monitoring technologies (Hardy et al., 2018; Schmidt et al., 2019; Yang et al., 2020).

Although many wished the device was smaller, several were satisfied with the big display and touch screen because of declines in visual acuity and dexterity. This suggests importance of a design that can be personalised according to an end user's capabilities and context of use (Hardy et al., 2018; Nguyen et al., 2017; Pateman et al., 2018). Moreover, many complained the device was uncomfortable and sweaty, potentially leading to behaviours that may impede smartwatch features (e.g. loosening a band) or avoidance of wearing the device under certain conditions (e.g. warm weather), which could affect data accuracy. Especially for improving adherence to intervention protocols in clinical studies, it is necessary to select a device allowing fewer barriers to adoption such as sweat interferences and skin problem exacerbation (e.g. interchangeable band or soft but durable plastics).

End‐user difficulties were found regarding complexity of using the location‐tracking feature. To turn the GPS function on, participants were forced to complete a sequence of steps to reach the activity navigation menu. These types of menu sequences are typical to reach location tracking on most smartwatches to map routes and calculate wearer speed and movement size. To maximise access these benefits, smartwatches should focus on different training and implementation strategies according to older adults' technology experience, understanding or literacy (Meppelink et al., 2015; Preusse et al., 2017). Also, researchers should consider development of easy‐to‐understand instructions combined with health literacy‐based techniques (e.g. teach‐back) to improve comprehension (Miller et al., 2011).

Many participants reported no concerns about privacy related to wearable activity trackers and location tracking. This may be because of the perceived benefits outweighing risks (Demiris et al., 2008) or participants' naivety about potential privacy risks and implications (Boise et al., 2013; Fausset et al., 2013). Because evaluations of the Fitbit app and data dashboard were outside the study aims and protocol, participants did not access these software interfaces and might have not been aware of possible sources of privacy violations, such as the electronic transmission of sensor data, data breaches and security risks. Lack of perceived privacy risks may influence smartwatch willingness to share personal activity and location data. Thus, privacy concerns reported by a few participants warrant further research related to location tracking‐related privacy to understand older adults' perceived risks of sensor‐based activity and location tracking.

Participants reported the potential usefulness of sensor‐based data for themselves, family members, and healthcare providers. They identified types of data they wished to see (e.g. heart rates, step counts, and calories burned), which seemed to be limited to the data types available through the display at the time. Some were dissatisfied with perceived capability of the device being limited to checking steps or heart rate. Making smartwatches more versatile and sensor data available to participants may increase the appeal of long‐term study participation and actual device use among older adults. Additionally, we found some wished to know what the values meant for their health outcomes (e.g. steps and calories). Therefore, it is important to transform sensor data into relevant and meaningful information for older adults, for instance, through visualisations, or to incorporate sensor data into a patient portal or electronic health records. It will ultimately support older adults' health‐related decision‐making and healthy behaviours based on the discovery of understanding of activity patterns and health status.

### 4.1 Limitations

This study has several limitations. First, due to the nature of the life‐space mobility monitoring protocol, participants used the device only for 3 days, during the daytime only. The purposely short period might have resulted in more initial impressions rather than factors leading to continued use of the device. Second, in considering the practical utility of our findings, we highlight the specifics of our participants. Those who were younger, more educated, and tech savvy may have been more strongly motivated to participate, which limited diversity of the sample. This study does not represent the perspectives of the ‘oldest old’. Third, the protocol precluded participant use of mobile apps related to activity data because a major study aim was to measure older adults' mobility in natural environments, and activity app use could potentially confound this measurement. Future research will examine the usability of fitness app platforms and perceived usefulness of data being displayed on the app.

## 5 CONCLUSION

Despite the growth in popularity of smartwatches with location‐tracking sensors, there is limited evidence regarding factors affecting the adoption and use of these devices among older adults. Smartwatches with location tracking have the potential to enable personalised detection and prevention of functional declines based on analysis of older adults' activities within one's life space. Our study provides early evidence regarding older adults' acceptance of GPS‐enabled smartwatches. Future research should be conducted over extended periods to assess changes over time in older adults' attitudes towards smartwatch location and activity tracking. Additionally, efforts should be undertaken to identify barriers and facilitators of smartwatch adoption and utilisation barriers to inform development of care models supported by wearable technologies and delivered to older adults with different levels of health status, technology experience and health literacy. Furthermore, to achieve equitable access to smart technology and wearables, there is a need for examining perceptions and use experiences among people of the Mature and the Silent Generations (Blazina & Desilver, 2021) who have had the least exposure to smartwatches over their lifetimes on average. This work will inform strategies for older adults' sustained adoption and use of GPS‐enabled smartwatches to capture changes in mobility levels.

## 6 Implications for practice

To support sustained self‐monitoring behaviours through wearable sensor devices, it is critical to examine how older adults perceive those devices and identify factors affecting technology acceptance that can maximize adoption.

## AUTHOR CONTRIBUTIONS

All authors critically reviewed the manuscript, performed significant editing and approved the final manuscript. JC designed this study and the concept of the analysis, conducted descriptive statistics and supervised the overall research processes. HRB collected data. JC and HRB analysed qualitative data and wrote and revised the manuscript. BR, OM and GD revised the manuscript for important intellectual content.

## CONFLICT OF INTEREST

The authors declare that there is no conflict of interest.

## Supporting information


Appendix S1

Table S1

Appendix S2

## Data Availability

The datasets presented in this article are not readily available because we do not have IRB approval to share the data. The data that support the findings of this study are available from the corresponding author upon reasonable request.
