# Supplementary material for: Community‐dwelling older adults' acceptance of smartwatches for health and location tracking
Source: Int J Older People Nurs. 2022 Jul 12;18(1):e12490. doi: 10.1111/opn.12490 (PMC10078487; doi:10.1111/opn.12490)
Supplement: Supplementary file 2 — Table S1 [file OPN-18-0-s001.docx]

Appendix Table 1. *Technology Acceptability Interview Guide*

| Interview Questions |
| --- |
| 1. **General Attitudes toward mHealth Technology-Based Monitoring**  - What do you think about the Fitbit you have used over the last three days?  Any concerns? [Cues: privacy, obtrusiveness, aesthetics] - On a scale of 1 (not at all) to 7 (very satisfied), how satisfied were you with the display of the device?   - Please describe why you selected that number. - On a scale of 1 (not at all) to 7 (very satisfied), how satisfied were you with the functions of the device?   - Please describe why you selected that number. - On a scale of 1 (being lowest) to 7 (being highest), how appealing was the device overall?   - Please describe why you selected that number.  1. **Perceived Usefulness and Perceived Ease of Use of the mHealth Technology**  - Would the data resulting from these sensors showing how active you are in your home and your living spaces have any usefulness for you?   - If yes, what kind of usefulness? - On a scale of 1 (being lowest) to 7 (being highest), how easy was it to use this device overall?   - Please describe why you selected that number.  1. **Access and Perceived Usefulness of the Sensor Data**  - Would you like to see the data from activity monitoring? - Would you share this data with your family? Health care provider? Others? - How often would you like to see the data about your own activities of daily living? (cue: mobility, dressing, eating, etc.)?  1. **Privacy and Other Concerns related to the mHealth Technology**  - Has having this device on your wrist changed how you carry out your daily activities?   - If yes, how has it changed the way you carry out your daily activities? - Would you like to be able to turn the device on and off depending on your preferences at any given time? - We have already touched upon privacy. Do you have in general any privacy or other concerns with the use of mHealth technology for health purposes? (examples: being monitored, inappropriate sharing, loss of human touch, maintenance issues, etc.) - Are there any other thoughts you would like to share about the system or this research study? |
