# Supplementary material for: Community‐dwelling older adults' acceptance of smartwatches for health and location tracking
Source: Int J Older People Nurs. 2022 Jul 12;18(1):e12490. doi: 10.1111/opn.12490 (PMC10078487; doi:10.1111/opn.12490)
Supplement: Supplementary file 3 — Appendix S2 [file OPN-18-0-s003.docx]

Appendix 2. Codebook

1. **Wearable Sensor Technology**

*This relates to the wearable sensor technology or idea of wearable sensor technology (capacity of Fitbit including GPS tracking).*

Responses to questions:

“What do you think about the Fitbit you have used over the last three days?”

“On a scale of 1 (not at all) to 7 (very satisfied), how satisfied were you with the functions

of the device?”

“On a scale of 1 (being lowest) to 7 (being highest), how appealing was the device overall?”

- 1. **Perceptions of Technology**

*Any comments about the understanding of technology functions, potential uses of the technology (e.g., if health declines, or prevention purposes) concerning general wearable sensor technology not specific to the Fitbit Surge*

1. *I liked it.*
2. *It has the potential to motivate me to move more.*
3. *I don’t need one to motivate me.*
4. *It would not change my activity level.*
5. *It was okay.*
6. *It would be useful.*
7. *It’s not useful.*
   1. **Factors affecting Technology Acceptance**

*Factors affecting technology acceptance (e.g., cost, living situation (living alone or with a partner), etc.)*

**c. Device Use dependent on Lifestyle**

*Any comments about lifestyle factors (e.g., swimming) that could affect the decision whether to purchase or use the technology or that could affect the decision regarding when to wear the technology*

1. **Device**

*This relates to the Fitbit itself (e.g., aesthetics, comfort, etc.).*

Responses to questions:

“What do you think about the Fitbit you have used over the last three days?”

“On a scale of 1 (not at all) to 7 (very satisfied), how satisfied were you with the display of the device?”

“On a scale of 1 (being lowest) to 7 (being highest), how appealing was the device overall?”

- 1. **Ease of Use**

*Comments about how easy it was to use.*

“On a scale of 1 (being lowest) to 7 (being highest), how easy was it to use this device overall?”

- 1. **Control**

*The ability to turn on/off.*

“Would you like to be able to turn the device on and off depending on your preferences at any given time?”

- 1. **Usability**

*Comments about function, display, satisfaction with the device, or effectiveness of the device to achieve goals*

- 1. **Aesthetic**

*Comments about how the device looks (e.g., ugliness)*

1. **Concerns**

*Any concerns or complaints about privacy, obtrusiveness, etc.*

Responses to questions:

“Do you have any privacy or other concerns with mHealth technology for health purposes in general?” (examples: being monitored, inappropriate sharing, loss of human touch, maintenance issues, etc.)

- 1. **Accuracy or function**

*Any concerns about how the device work (e.g., is it really capturing what it is supposed to capture?). This is differentiated from general comments about the device's function in 2.2. Usability*

- 1. **Behavior change**

If a participant reported his/her behavior was changed due to a concern of being monitored or a feeling that their privacy was invaded (*“I didn’t want to be tracked, so I didn’t go as many places.”).*

“Has having this device on your wrist changed how you carry out your daily activities?”

- 1. **Potential harm to health**

If a participant reports a concern related to the potential effect of the device on health (e.g., blinking light of the device feels like radiation)

- 1. **Privacy**

*Any concerns related to privacy invasion due to the device use or sensor data*

- 1. **Security**

*Any comments related to the potential threat to the protection of personal data; possible examples of reasons causing this situation include theft, the device left unattended, or hacking.*

- 1. **Obtrusiveness**

Any comments on whether the device is noticeable or not; how the obtrusive feature affected a participant’s feeling or the way they carry out activities or interact with other people

1. **Data Use**

*Any comments about potential usefulness of personal activity data in general and for others and desire to access their individual activity data if the data were made available to them.*

- 1. **Desire to access data**

*Any comments about an expressed desire to access/look at their activity data if the data were made available to them*

- - 1. ***Desired frequency of data access***

*Answers to the question “How often would you like to see the data about your activities of daily living?”*

- 1. **Perceived usefulness of data**

*Any comments about whether the data resulting from the device showing how active the participant has any usefulness; this is related to the data, not technology.*

- 1. **Specific uses of data**

*Comments about when or for what reason they would want to use their data*

- 1. **Types of data interested**

*Specific types of data that are shown either on the device or presented to participants as a form of summary data*

- 1. **Willingness to Share Data**

*Reasons for sharing data or not sharing data. Any comments about one’s willingness to share his/her own data with others. Any related concerns.*

Responses to questions:

“Would you share this data with your family? Health care provider? Others?”

- - 1. Reasons for sharing
    2. Reasons for not sharing
    3. Sharing with family
    4. Sharing with health care provider(s)
    5. Sharing with others

1. **Physical activity and mobility**

*Comments about physical activities and mobility (e.g., “I thought I walked four miles a day, but this says I only walked one.”).*

- 1. **Activity level change due to device use**

*It could be increased or decreased physical activity or other behavior change because of Fitbit use (e.g., “This motivated me to exercise more”).*

“Has this device on your wrist changed how you carry out your daily activities?”

- 1. **I do enough exercise**

*If a participant thinks that they do enough level of exercise in daily living*

- 1. **I don’t move around enough**

*If a participant thinks that they do NOT do enough level of exercise in daily living*

- 1. **Realization**

*If a participant mentions that the device use made them realize how they are active or what the actual level of physical activity is*

1. **Other**

*Anything mentioned that might be useful, but doesn’t fit with another code.*

“Are there any other thoughts you would like to share about the system or this research study?”
